# Lightweight Concrete—From Basics to Innovations

**DOI:** 10.3390/ma13051120

**Published:** 2020-03-03

**Authors:** Karl-Christian Thienel, Timo Haller, Nancy Beuntner

**Affiliations:** Institute for Construction Materials, Universität der Bundeswehr München, Werner-Heisenberg-Weg 39, 85579 Neubiberg, Germany; timo.haller@unibw.de (T.H.); nancy.beuntner@unibw.de (N.B.)

**Keywords:** lightweight concrete, lightweight aggregate concrete, infra-lightweight concrete, LC, LAC, ILC, lightweight aggregate, LWA, production, mix design

## Abstract

Lightweight concrete has a history of more than two-thousand years and its technical development is still proceeding. This review starts with a retrospective that gives an idea of the wide range of applications covered by lightweight concrete during the last century. Although lightweight concrete is well known and has proven its technical potential in a wide range of applications over the past decades, there are still hesitations and uncertainties in practice. For that reason, lightweight aggregate properties and the various types of lightweight concrete are discussed in detail with a special focus on current standards. The review is based on a background of 25 years of practical and theoretical experience in this field. One of the main challenges in designing lightweight concrete is to adapt most of design, production and execution rules since they often deviate from normal weight concrete. Therefore, aspects are highlighted that often are the cause of misunderstandings, such as nomenclature or the informational value of certain tests. Frequently occurring problems regarding the mix design and production of lightweight concrete are addressed and the unintended consequences are described. A critical view is provided on some information given in existing European concrete standards regarding the mechanical properties of structural lightweight concrete. Finally, the latest stage of development of very light lightweight concretes is presented. Infra-lightweight concrete is introduced as an innovative approach for further extending the range of applications of lightweight concrete by providing background knowledge and experiences from case records.

## 1. Introduction

Lightweight concretes are not a modern achievement of concrete technology. They have been known since ancient times and are basically the predecessors of today’s concrete. The first European references of lightweight concrete were built two thousand years ago during the early Roman Empire. The Pantheon in Rome, Italy, was built ca. 128 A.D. and can be cited as one of the best-known examples. It has amazed engineers from various disciplines over hundreds of years and has impressively demonstrated the systematic use of various natural lightweight aggregates in opus caementitium [1]. After the collapse of the Roman Empire, the use of lightweight concrete was limited due to the low availability and variability of natural, volcanic aggregates. The development and production of industrially produced lightweight aggregate in the 19th and 20th centuries marked a historic turning point for material technology [2,3]. 

Initially, the use of the expanded aggregates was reserved for the Navy of the United States of America. The associated U.S. Emergency Fleet Building Corporation established a shipbuilding program with the U.S. entry into World War I. In 1918, Atlantis was the first ship to emerge from this program and fourteen lightweight concrete ship hulls were built in total. During World War II, these early experiences led to the deployment of 104 supply ships, with cargo capacities ranging from 3200 to 140,000 tons [4]. 

Its successful use in shipbuilding has enabled the introduction of lightweight concrete in structural engineering. The first commercial plant to produce expanded aggregates has been established in 1920 in Kansas, USA. The uniform quality and composition of the industrially produced aggregates have proved to be advantageous over aggregates from natural origin [3].

During the 1920s, several bridges were built using expanded slate as aggregate in lightweight concrete. Fifty years later, more than 200 lightweight concrete bridges have been constructed in the United States and Canada [5]. In the middle of the 20th century, the use of lightweight concrete in structural engineering intensified. It led to multi-story high-rise buildings such as the Prudential Plaza Building or the Marina City Towers in Chicago. However, lightweight concrete was predominantly used for structural reasons and a conscious adaptation of lightweight concrete by the architecture remained largely limited to individual cases [6]. 

As consequence of the oil crisis in 1973, Germany has reconsidered its political position with regard to energy consumption. In order to become more independent of energy imports, the Energy Saving Act [7] was implemented as a logical consequence. Among other things, the efficient use of energy in buildings has become a political obligation. As one of the consequences, monolithic exterior walls made of lightweight concrete with an economical thickness no longer met the increased thermal insulation requirements.

Since the 1990s, the technical regulations for building with concrete in Germany have been revised. The use of lightweight concretes has experienced an upswing due to these new regulations, as well as the ongoing developments in both technology and science. However, the number of examples carried out remained comparatively low. Recent developments aim on a further reduction of the concrete density, while maintaining a sufficient and as high as possible strength. Within the last years, monolithic structures made of fair faced lightweight concretes in the lowest strength and density classes have gained popularity in Europe. These structures provide the construction of a finished wall in one operation and, thus, prevent the use of multi-layer wall structures [6]. The decision in favor of a massive lightweight concrete wall is usually based on the desire to design an individual architecture that can be shaped easily, while exploiting the design possibilities of exposed concrete. Moreover, the physical properties of lightweight concrete, such as low density, favorable building physics and high fire resistance are the main characteristics of a promising material [8,9,10,11] as well as its excellent durability [12,13,14,15,16,17].

The latest stage in this development includes the so-called infra-lightweight concrete (ILC) [18]. Other researchers refer to similar material approaches under terms like Warmbeton [19], Architekturleichtbeton [20] or ultra-lightweight concrete [21]. ILC aims at fulfilling both structural specifications and thermal insulation requirements. ILC offers possibilities for monolithic wall design, meeting current regulations for energy consumption in buildings without using additional insulation material. Despite the technological point of view, building regulations are still a challenge for the application of ILC in practice. ILC is not yet a standardized building material and thus far, it is necessary to obtain a project-related or a technical approval. However, it is possible to order ILC commercially.

This paper focuses on structural lightweight concrete (LC) based on mineral lightweight aggregate (LWA). The basic constituents of LC, their interactions and influence on mechanical properties and durability can differ significantly from normal weight concrete (NC). This justifies increasing attention and the necessity to adjust most of the design, production and execution rules compared to NC. Therefore, this work provides a basic overview of the main constituents, their properties and the associated special features in mix design and production. Frequently occurring problems, missing or questionable information in some European standards are highlighted. In addition, this paper gives an outlook on the latest developments in the field of ILC. The aim of this publication is to consolidate the basic understanding of design criteria, to promote a better understanding in the practical application of lightweight concrete and to establish ILC in construction practice.

## 2. Description of Lightweight Aggregate and Lightweight Concrete

### 2.1. Lightweight Aggregate

Lightweight aggregates (LWA) are specified in international standards like EN 13055 [22], ASTM C330M [23], ASTM C331M [24], and ASTM C332 [25]. The ASTM standards distinguish between LWA for structural lightweight concrete [23], LWA for the application in masonry lightweight concrete [24] and LWA for insulating concrete [25]. The European standard EN 13055 holds amongst others for LWA to be used for any type of lightweight concrete. The standards mentioned, however, do consider only LWA of mineral origin. EN 13055 lists LWA not by their common name, but defines their sources. LWA can be of natural origin, manufactured from natural materials, manufactured from by-products of industrial processes or from recycled source materials, or by-products of industrial processes [22]. The ASTM standards have an explicit list of LWA covered: “Two general types of lightweight aggregates are covered by this specification: aggregates prepared by expanding, pelletizing, or sintering products such as blast-furnace slag, clay, diatomite, fly ash, shale, or slate; and aggregates prepared by processing natural materials, such as pumice, scoria, or tuff” [23]. In addition, lightweight “aggregates consisting of end products of coal or coke combustion” are listed in ASTM C331M [24]. Literature, e.g., [26,27,28,29], provides details about the various types of LWA, their properties and typical production processes.

Besides their origin, the definition of the aggregate properties, mainly the density, is important in order to distinguish between normal weight and lightweight aggregate. ASTM C330M [23] and ASTM C331M [24] give upper limits for the loose bulk density 1120 kg/m^3^ for fine LWA, 880 kg/m^3^ for coarse LWA and 1040 kg/m^3^ for the combination of fine and coarse LWA. Additionally, it should be possible to produce structural lightweight concrete with LWA conforming ASTM C330M as given in Table 1, where compressive strength is determined on cylindrical specimens [30].

The possible range of LWA types and applications is further defined in EN 13055. Restrictions apply to loose bulk density (ρ_s_) (≤1200 kg/m^3^) and particle density (ρ_k_) (≤2000 kg/m^3^). Thus the standard covers even very light LWA like expanded perlite and exfoliated vermiculite which both are rather used in mortar and plaster or for “concrete not exposed to the weather, in which the prime consideration is the thermal insulating property of the resulting concrete” [25].

The use of any other LWA than the ones given above requires project-related or technical approval before being considered for any standardized lightweight concrete in practice or is limited for research purpose only. This holds, e.g., for cold-bound LWA [31] or any organic material like wood chips, rubber crumbs, plastic beads, or expanded polystyrene (EPS). Another major field of interest includes the use of recycling products as LWA. The potential base materials, such as rice husk ash [32], dredged silt [33], and polyethylene terephthalate waste [34], are some examples of the numerous possibilities. The following text will consider only lightweight concrete produced with LWA covered by the aggregate standards mentioned [22,23,24,25].

The crushing resistance according to EN 13055 [22] is often used to select or compare coarse LWA for lightweight concrete. It has to be noted that this approach ignores completely the background of the crushing resistance test: it is intended for production control only and shall provide information for conformity control. This test method is originally part of a Soviet standard [35]. The crushing resistance is determined at a compaction of 20%, while in concrete the maximum strain in compression is 3.5 mm/m. Thus, using this test for any other purpose than conformity control is a crude misunderstanding of the underlying principles and the intention of the test. A note in EN 13055 points out that “there is no simple relationship between the crushing resistance of lightweight aggregate and the properties at its end use” [22].

### 2.2. Lightweight Concrete

There are several definitions of lightweight concrete, which often leads to a lack of precision when referring to lightweight concrete. Deviations exist for strength, density and the type of lightweight concrete covered. ACI 213R-14 “Guide for Structural Lightweight-Aggregate Concrete” [36] specifies a minimum cylinder strength of 17 MPa and an equilibrium density between 1120 and 1920 kg/m^3^ for structural lightweight concrete (SLC) and without any strength requirement an equilibrium density between 800 and 2240 kg/m^3^ for specified density concrete (SDC). SLC with compressive strength of 40 MPa at 28 days is classified as high strength lightweight concrete.

**Figure 1:**
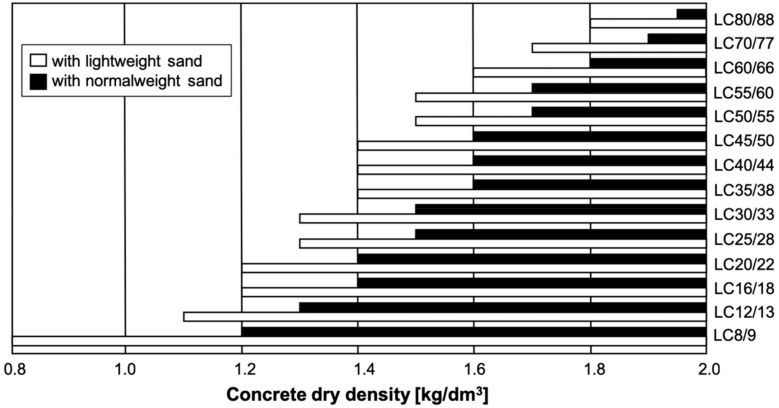
Correlation between strength classes and necessary dry density for LC [40,41].

**Figure 2:**
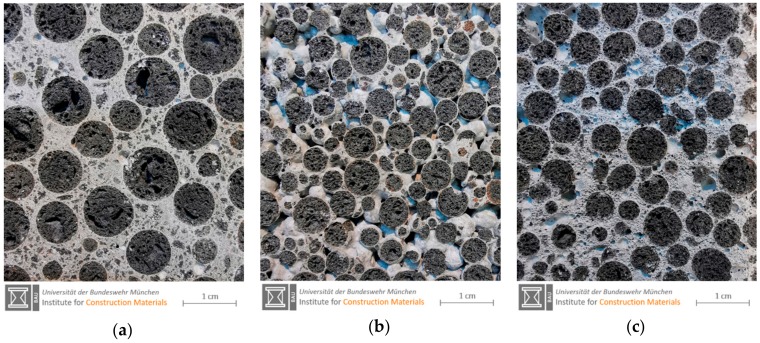
(**a**) Image of structural lightweight concrete (LC) with a dense matrix structure; (**b**) Image of the lightweight aggregate concrete (LAC) with open pore structure; (**c**) Image of LAC with a porous matrix filling the open pore structure. All three types of lightweight concrete are based on expanded clay from one producer as LWA.

In Europe, structural lightweight concrete (LC) is covered as material in EN 206 [37] and its application is regulated in EN 1992 [38]. Minimum strength class is LC8/9 referring to a characteristic cylinder strength of 8 MPa and a characteristic cube strength of 9 MPa [37]. The design standard [38] requests a minimum strength class of LC12/13 [37]. Due to a lack of practical experience, a technical approval is mandatory in Germany for strength classes LC70/77 and LC80/88. LC has an oven dry density of 800 ≥ ρ_d_ ≤ 2000 kg/m^3^. The density range is divided into density classes with a span of 200 kg/m^3^. A free and unrestricted combination of strength and density classes is not possible [39]. However, Figure 1 shows that specific LC strength classes require certain density classes (D) for a proper definition. Compared to other types of lightweight concrete, LC has a dense cement matrix and its surface can hardly be distinguished from normal weight concrete (NC). The lightweight aggregates become visible only on a damaged or cut surface (Figure 2a).

Care should be taken when referring to terms like lightweight aggregate concrete (LWAC), all-lightweight aggregate concrete (ALWAC) or sand lightweight aggregate concrete (SLAWC) since these terms are not specified in standards and often subject to individual definitions. Hence, any reference to such unspecified definitions may result in an incorrect or at least an incomplete statement.

Lightweight aggregate concrete with open structure (LAC) differs significantly from the aforementioned lightweight concrete (LC). Its properties are defined in EN 1520 [42]. The dry density of LAC ranges from 400 ≥ ρ_d_ ≤ 2000 kg/m^3^. EN 1520 covers strength classes LAC 2 to LAC 25, which are based on the characteristic strength [MPa] of 100-mm cores drilled from LAC elements. LAC is used for structural elements like loadbearing walls, roof elements, slabs and beams, and for non-structural components like noise barriers. Some properties of LAC can be improved significantly when the open structure is filled with a porous matrix (aerated cement paste) [43]. Figure 2 gives an idea of two different LAC types in comparison with a structural LC. LAC is characterized by defined voids between the aggregates that remain in the structure after compaction (Figure 2b). These voids are created by limiting the cement paste content to the amount required for binding the aggregates at the points of contact. There is no standardized definition regarding the minimum pore volume threshold to consider a concrete as "open porous". A planned pore volume of about 10% by volume can be assumed, which is the upper limit for structural concrete when adding air-entraining agent (LP concrete) [44]. Hence, the distinction must be made on the basis of the volumetric mix design.

A recent kind of lightweight concrete was developed initially for an application outside building industry. This very light lightweight concrete was intended for sandwich ship hulls. The underlying concept for design of ships and marine structures was developed by using sandwich plates composed of steel skins with a very low density lightweight concrete as core material [45]. Schlaich adopted this type of lightweight concrete and established infra-lightweight concrete as a non-standardized lightweight concrete [18,46]. ILC has a dry density of less than 800 kg/m^3^. Its compressive strength is less or equal than LC8/9 [47,48]. ILC provides a fair faced concrete surface and enables monolithic external concrete walls without additional insulation. Its structure after compaction rather complies with a LAC with a porous matrix (aerated cement paste) (Figure 2c) than with a LC. Thus far, the standards for structural concrete [37,38] have been applied for the design of ILC. In doing so, the design rules were extrapolated towards lower densities. This could be accomplished with a project-related approval [48].

New approaches are now initiated at Universität der Bundeswehr München in cooperation with several industrial partners. The basic idea is combining the initial design concept of ILC with the technical regulations laid down in EN 1520 [42]. The actual challenge is establishing rules for conformity on site. This is already part of a first project-related approval [49] and shall be transferred into a European technical approval (ETA).

## 3. Constituents, Mix Design and Production of Lightweight Concrete

### 3.1. Other Constituents in Lightweight Concrete than LWA

#### 3.1.1. Normal Aggregate

Most LC use a combination of lightweight coarse aggregate and normal weight sand. The decision depends among others on the requirements regarding specified strength and density, thermal conductivity and the market price of the aggregates. The quality and availability of LWA gradings are further factors that influence the optimum ratio of normal weight aggregate and LWA. Especially fine LWA often are available as crushed material only, resulting in a high water demand and affecting workability characteristics. The requirements for normal weight sand are the same as for their use in NC. 

#### 3.1.2. Binder Materials

LC can be produced with any cement available. For LC made with LWA it is highly recommended to use a cement with a modest specific heat release, like blast furnace cement, or cements in combination with fly ash, calcined clay, granulated blast furnace slag or limestone. These LC have a low thermal conductivity which could lead to high temperatures in the core of the construction element [50] during hydration exceeding the critical limit for delayed ettringite formation at 60 °C–70 °C [51]. The addition of SCM is discussed controversially. Demirboga and Gül [52] observed a reduction of strength when using microsilica and fly ash which also reduced thermal conductivity by about 15%, while Chung et al. [53] found improved mechanical and thermal properties due the use of fly ash or limestone powder. Shafigh et al. [54] investigated the addition of fly ash and limestone powder as well and noted that the latter improved the compressive strength of lightweight concrete in the early and late ages. Abd Elrahman et al. [55] performed experimental investigations on the influence of nanosilica, which improved strength and transport properties significantly. 

#### 3.1.3. Water

Any kind of water used in normal concrete production can be used for LC as well. This includes recycling water [56].

#### 3.1.4. Admixtures

Any admixture used in normal concrete production can be used for LC as well. The compatibility with the binder and LWA used should be checked beforehand, as for NC. This holds especially for LWA that may have different surface charges compared to normal weight aggregate. Initially dry or only prewetted LWA will absorb parts of liquid admixtures if these are added too early to the mix (see Section 3.2). ACI 304R-00 [57] recommends the use of presaturated LWA to avoid absorption of the admixtures into the LWA. Delayed addition of liquid admixtures will reduce the problem [26].

### 3.2. Producing LC

#### 3.2.1. Mix Design of LC

The mix design for LC differs fundamentally from NC due to the dominant impact of the LWA used [55,58,59,60,61,62]. Proportioning of LC must consider the boundary conditions like strength, density and durability, but also takes into account the casting situation and equipment. Detailed considerations can be found in [36]. When designing for a certain strength, a coarse LWA must be selected which has sufficient strength capacity. In the case of doubt local LWA producers should be asked for their advice [36]. The strength of selected coarse LWA sets the ceiling for the LC strength. The threshold above which the LWA is decisive and the strength of the matrix becomes a secondary factor for the potential LC strength is called strength limit (see Section 4.2) [62,63,64]. Above this strength limit, binder content and water to binder ratio play a secondary role in LC. However, they also determine the durability properties of LC and thus have to be chosen properly (e.g., according to DIN 1045-2 [65]). The type of sand has a major impact on thermal conductivity. Replacing normal weight sand with lightweight sand reduces in a typical LC the dry density by approximately 200 kg/m^3^. At the same time, compressive strength is lowered as well depending on the type of lightweight sand selected. 

**Figure 3:**
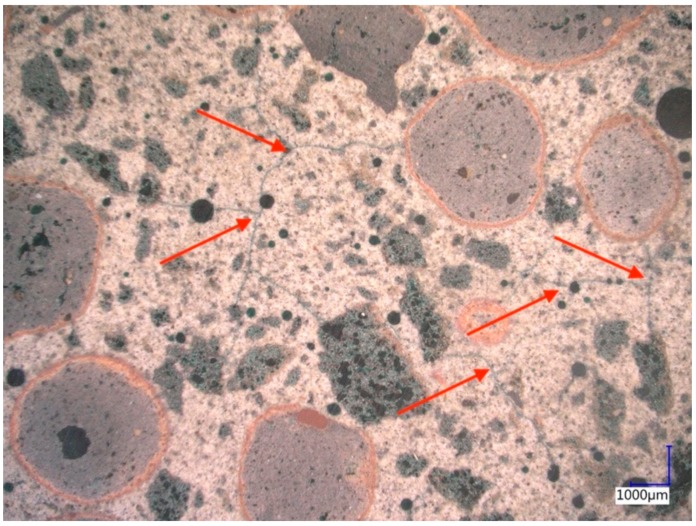
Severe formation of microcracks in the matrix (marked with red arrows) of a high strength LC due to water absorption of fine and coarse LWA (Courtesy: Andrea Kustermann).

Mix design of LC should always disclose the water added to account for the water absorption of the LWA, although the water will not increase the LC volume [66,67]. The w_60_ value indicates the water absorbed by coarse LWA within 60 minutes and is a common parameter in practice [68]. This experimental value, however, does not respect the actual moisture stage of the coarse LWA, which in turn has an impact on the effective water absorption [66,69,70]. It is even more important to account for the high water absorption of fine LWA. It ranges from 25 to 40 wt% [27] and must be considered in mix design at least to a certain extent. Thus far, there exists only one proposal related to this topic [40] which recommends to consider 70% of the water absorption measured according to DIN V 18004 [71]. This test method will be adopted in the next version of EN 1097-6. Ignoring the water absorption will cause an uncontrolled uptake of water from the matrix by the LWA and especially by the lightweight sand. This will consequently lead to the formation of serious microcracks in the matrix (Figure 3) which reduce the achievable strength and jeopardize the durability. Since the absorption takes place during the plastic stage of the LC matrix, it cannot be compensated by later internal curing effects [72].

Any mix design for LC must be given in dm³/m³ based on volumetric share of the constituents. The weight is an inaccurate measure for LC. Nevertheless, this weight information shall be provided together with the particle densities used in the mix design. By doing so, the air void content must be given as well. It is often missing in mix designs. Measurements of the air void content following ASTM C 173 [73] must always be double-checked considering the yield [74] and the density achieved. Other methods [75] will not provide correct information about the air void content since one cannot distinguish between air voids in the paste and air entrapped in the LWA. This method can be used on site for conformity control. 

#### 3.2.2. Mixing and Delivery of LC

The dosage of the LWA should be volumetric wherever possible. Since in most concrete plants gravimetric weighing is available only, the moisture content and absorption of the LWA [69] as well as their loose bulk density must be checked at appropriate intervals and changes taken into account to adjust the dosing. All other components are measured as usual.

During mixing, the LWA are filled in first. Up to two thirds of the required mixing water and the water compensating the absorption are added to the running mixer and mixed in for about 30 seconds. This is particularly important when lightweight sand is used as it absorbs a relatively large amount of water. If LWA is prewetted, it should be as uniform as possible [36]. Next, the cement is given into the mixer, followed by the remaining mixing water. Powdery additives are added along with the cement. If silica fume is used, it should go either as powder with other dry additives or as slurry together with the remaining mixing water. As mentioned beforehand in Section 3.1.4, admixtures should be added as late as possible and in the best case with the remaining mixing water to prevent uncontrollable absorption into LWA. The minimum mixing time of structural lightweight concrete, after the addition of all constituents, should be prolonged from a range of 30 to 60 s up to 90 s, compared to NC.

Structural lightweight concrete is preferably produced in a compulsory mixer. If possible, the mixer blades should have a plastic lining in order to avoid unnecessary crushing of the LWA, especially in the case of very light LWA. In concrete plants, up to 5–10% of the very light coarse LWA can be crushed. In the laboratory, this grain fragmentation is between 3% and 5%, in some laboratory mixers also significantly higher. Water absorption of LWA and LC density both rise due to the LWA fragmentation. Workability will be reduced due to the higher water absorption of the crushed LWA.

The fresh LC should be checked for unit weight and yield [74]. One option is the comparison of the fresh density with the density in the mix design. A second method requires an accurate job in the laboratory. The effective volume of the trial batch should be verified. When compacted, the combined volume achieved can be checked against the intended volume. The initial mix design must be corrected by the yield factor. Due to all the uncertainties, the yield may vary by ±5 to ±10% by volume for LC. The latter holds for LC made with coarse and fine LWA. Here, the tests available for particle density of fine LWA [68,71] will provide non-suitable values since they do not account for the real water absorption in a mixer [76].

As a result of the water absorption of the lightweight aggregates, the duration of transport and placing has a greater impact on the workability of LC than on that of NC. The degree of compactability [77] should be used to assess the workability and compare different LC. The values of the flow table test [78] can be misleading, as the weight of the aggregates acts as the main driving force in this test. With LC, different flow consistencies can therefore be determined for apparently identical workability, depending on the density of LWA used.

#### 3.2.3. Placing and Handling of LC

Structural lightweight concrete does require the same placing techniques as used for NC [36]. LC is mainly placed on the construction site by using buckets, which is one reason for its restrained use. In the USA, however, the pumping of LC is common practice thanks to the use of water-saturated LWA. The obvious advantage comes along with higher density and increased transport costs. In the meantime, technology of LC in Europe has progressed so far that even LC with only pre-wetted LWA, can be pumped accurately [41]. 

When pumping LC with non-water saturated LWA, the water in cement paste is pressed out by the pumping pressure and ingresses together with fine particles into the pores of air-filled LWA [79]. This, in turn, compresses the volume of LC and as consequence, the consistency of LC declines due to the reduced water content left in the paste. As soon as the pressure on the fresh LC drops at the end of the pump line or—depending on the design of the pump—in the pump line, the air within the LWA pores and the LC itself can relax and expand. The air trapped in the pores of the LWA forces again the water out of the LWA. This often leads to segregation effects of the LC and can indicate pumping fails. Such behavior can even occur despite a previous, longer lasting (e.g., 24 h) water storage of the LWA. It can be remedied by specially adapted, almost self-compacting LC mix designs. A suitability test including a pumping test is always recommended before pumping on construction sites.

Even if the pumping of LC has been successful, it may have negatively affected the quality of the hardened concrete [69]. The cause is again the water squeezed into the pores of the LWA during pumping. After relaxation, the water is displaced by the air, which was beforehand trapped and compressed inside the aggregate during the pumping process. As a consequence, a water seam forms around the LWA and a porous ring remains after hardening. A comparable effect can occur if the LC is not remixed at the construction site before placing [80].

### 3.3. Mixing and Delivery of ILC

Infra-lightweight concrete combines LC with several ideas used for LAC with a porous matrix. Thus, the mix design approach is similar as for LC. In order to reduce density and thermal conductivity, the air void content exceeds the limitation of 10% by volume for LC [44]. The necessary workability for placing this concrete on site is maintained by adding sufficient air entraining admixture, without jeopardizing the intended fair faced surface quality [18,48]. For mixing, transport, and placing, the same rules apply as for LC. Pumping will most likely destroy the air void system and was thus far omitted in all executed projects.

## 4. Microstructure of LC and Resulting Consequences

### 4.1. Interface between LWA and Matrix

**Figure 4:**
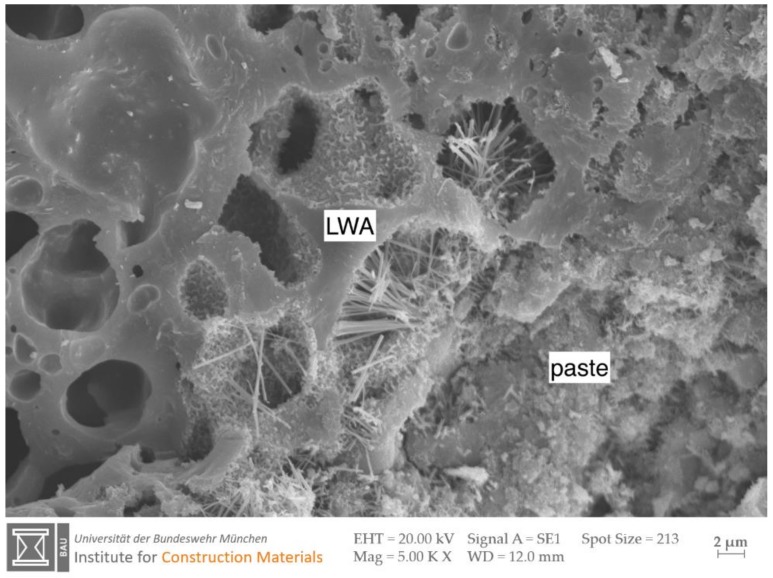
SEM image of the interfacial transition zone in lightweight concrete between an expanded clay aggregate and the paste. Hydration products are visible inside the outer LWA pores [97].

The microstructure of LC differs significantly from that of NC. The latter represents a 3-phase system of aggregate, matrix and the interfacial transition zone (ITZ) around the aggregate [81,82]. In contrast to NC, there is no ITZ in LC when correctly composed (Figure 4). This is due to four mechanisms:

1. The LWA absorbs water during mixing. Together with the mixing water, parts of the binder components infiltrate the porous LWA. The hydration products therefore do not only grow towards the outer LWA surface, but also to a limited extent towards the inside of the LWA (Figure 4). The resulting increase in particle strength is associated with an increase in bulk density and a loss of binder component in the matrix. The intrusion of binder components into the LWA provides an advantage, but it is more economical to keep them in the matrix [83].
2. Some expanded clays exhibit reactive clinker phases, such as Gehlenite (C_2_AS), on the outer shell of the coarse aggregate. These LWA can therefore react with the binder components to a limited extent [84]. With regard to reactive LWA surfaces, there is greater research interest in the impact of cold-bound aggregates [85] as mentioned above (see Section 2.1), as well as in LWA synthesized by cementing and geopolymerization [86].
3. The LWA surface is rough and porous and permits very good mechanical interlocking [29].
4. As mentioned beforehand, the water absorbed by the lightweight aggregate is available for optimum internal post-treatment in the course of the hydration. This property is partly used in high strength normal concretes [87,88,89].

The absence of a pronounced ITZ affects load-bearing behavior and durability [15,90,91,92,93]. It is particularly important for the composition and mixing of LC when silica fume is part of the mix design. In high strength NC, the addition of silica fume is intended above all to improve the ITZ (e.g., [81,94,95,96]). In order to bring it to the surface of the normal weight aggregate of NC, the silica suspension is added to the mixer immediately after the aggregate has been added to the mixer. In LC, the silica suspension would partly be lost in the LWA and is therefore added later to the mix (see Section 3.2.2) to improve the quality of the matrix.

### 4.2. Load-bearing Behavior of LC

The strength of the mortar matrix determines the strength of NC. Therefore, a uniform relationship between the concrete and matrix compressive strength can be established. Mortar strength is characterized by the w/c ratio and the standard compressive strength of the cement.

In the case of LC, the LWA are often less solid and less stiff than the matrix, depending on the density of the LWA. The LWA is therefore decisive for the strength of the lightweight concrete structure. As a result, the concrete strength may lag behind the compressive strength of the matrix [29,98].

At young age, the strength of LC and NC initially develops simultaneously [99,100]. Under compressive loading, the main compressive stress trajectories run in a concentrated manner from coarse particle to coarse particle. The mortar layers transmit the compressive forces and are predominantly subjected to compressive loading. At right angles to the direction of loading, tensile stresses arise due to the deflection of the compression trajectories. This, in turn, does also stress the adhesive bond between aggregate and matrix. As in NC, adhesive cracks and finally fracture occur below a limit strength that depends on the LWA properties [100,101].

As hydration progresses, the stiffness and strength of the matrix can exceed that of the LWA. In this case, the internal flow of forces changes [29,100]. The main compressive stresses run around the LWA. The mortar layers are subjected to compressive stress. Tensile stresses arise above and below the LWA at right angles to the compression trajectories, i.e., approximately at right angles to the direction of loading. If the tensile stresses exceed the matrix tensile strength, cracks form there. The tensile forces are gradually transferred to the LWA until their tensile strength and thereby finally the strength capacity of the LC is reached. The threshold above which the LWA strength capacity becomes the strength determining factor for LC is known as strength limit (f_lc,lim_) [29].

**Figure 5:**
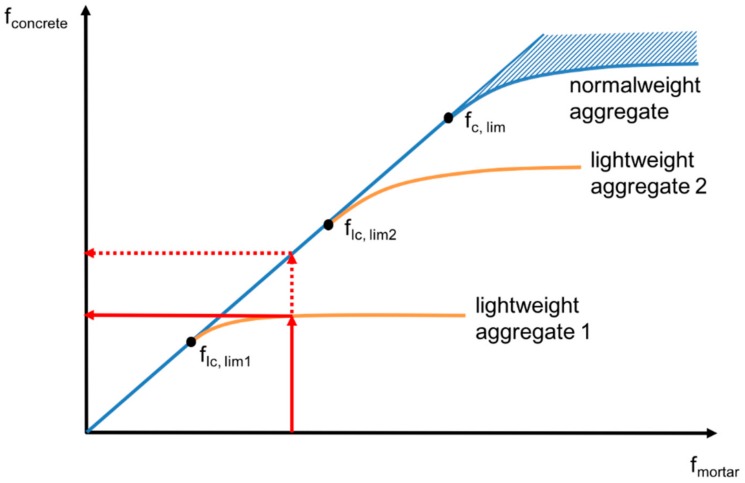
Explanation of limit strength (f_lc,lim_) of different types of lightweight and normal weight aggregate [64].

Up to a level, which dependents on the strength capacity of the LWA, strength of LC does more or less coincide with the matrix strength to a certain level. Above this level, the strength of LC increases disproportionately less with an increase in matrix strength [102,103]. In Figure 5, “lightweight aggregate 1” could for instance represent an expanded clay with ρ_s_ ~ 350 kg/m^3^ and “lightweight aggregate 2” an expanded clay with ρ_s_ ~ 600 kg/m^3^.

Since the lightweight aggregates contribute increasingly less to the load transfer above the limit strength, the matrix strength must be drastically enhanced to further increase the concrete strength. This procedure is not economically meaningful and it is better to select a stronger LWA instead. The ultimate LC strength depends on the LWA used: The stronger the LWA, the higher the ultimate strength of the LC. 

For a mortar strength below the limit strength, the strength of LC differs only slightly from the matrix strength, i.e., it corresponds approximately with that of a NC of the same strength. This is important with respect to strength classes and conversion factors (see Section 4.3). The ultimate strength is reached when stiffness of the matrix exceeds that of the aggregate, so that the grains participate increasingly less in the transmission of forces according to their volume fraction [29].

### 4.3. Conversion Factors for LC

#### 4.3.1. Conversion Factors for Different Specimen Sizes and Shapes

**Figure 6:**
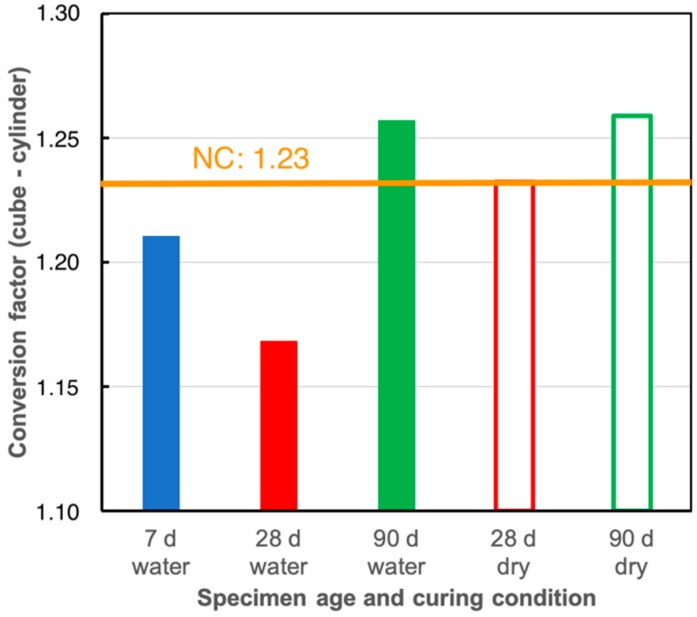
Conversion factors between 150-mm cubes and 300/150-mm cylinders for LC tested at 7 d, 28 d and 90 d. The specimens were cured under water [116] or in a climate room (20 °C/65% relative humidity) following the German national annex to [116] [64].

The load bearing behavior of LC has an impact on conversion factors applied for different specimen shapes and sizes [104,105,106]. The cylinder strength is always considered being the reference for uniaxial compression [36,38]. Nevertheless, cubes are the most common and sometimes the only test specimens in many European countries, e.g., Austria [107], Belgium [108], Germany [109], The Netherlands [110], and U.K. [111]. Originally, standards always used identical conversion factors for NC and LC. For the first time Model Code 90 [112] assumed the possibility of deviating factors for LC and NC. “If national standards require different types of specimens or different storage conditions up to the time of testing, conversion factors which have been verified by tests have to be applied.” These deviating conversion factors are based on the recommendation given in [113]: “For LWAC the cylinder strength shall be the only reference strength. The cube strengths specified in Table 2.1.1 [of Model Code 90, the authors] are thus not valid.” To the best knowledge of the authors, the tests recommended have not been performed. With the introduction of EN 206 and EN 1992, the conversion factor between 15-cm cubes and 30/15-cm cylinders was 1.23 for NC and 1.10 for LC. The preceding Section 4.2 on load-bearing behavior of LC pointed out the difference between the behavior of LC below and above the limit strength (f_lc,lim_). For economic reasons, most LC in practice will have a design strength below f_lc,lim_. Thus, their behavior is dominated by the matrix as it is the case for NC and the conversion factors between different specimen sizes are expected to be the same for LC and NC. This was confirmed in a series of tests involving more than 200 mix designs with various types of LWA, covering the strength range between 15 and 70 MPa and densities between 950 and 1700 kg/m^3^ [64,114]. Figure 6 gives the resulting conversion factors between 15-cm cubes and 30/15 cm cylinders for different testing ages and curing conditions. These and other results [115] cast severe doubts to whether a deviating conversion factor between cylinders and cubes for LC is reasonable and correct. The actual conversion factor in [37,38] for LC (i.e., 1.10) may lead to a faulty anticipation of the cylinder strength, and in some cases to an incorrect declaration of strength classes, if cubes are used as only specimen type [64,114].

#### 4.3.2. Conversion Factors for Different Curing Conditions

**Figure 7:**
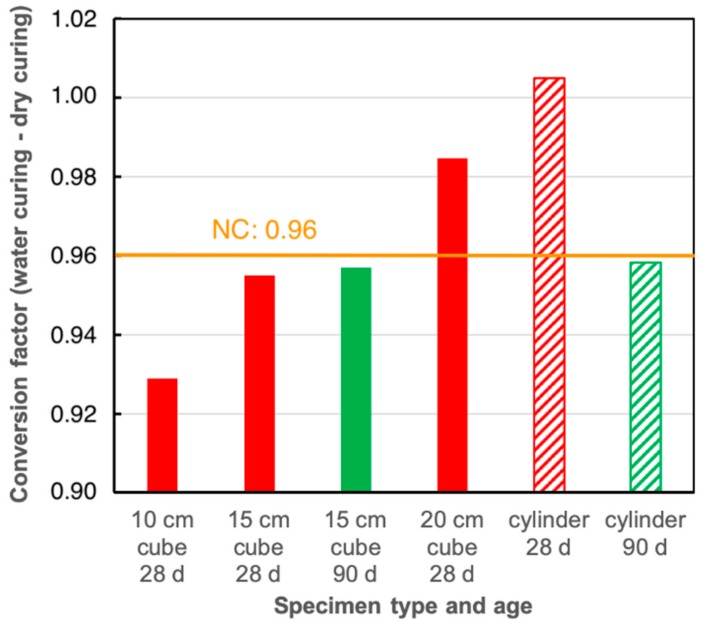
Conversion factors between specimens cured under water [116] or in a climate room (20 °C/65% relative humidity) following the German national annex to [116] for different specimens sizes, shapes and testing ages [64].

Figure 7 shows conversion factors between various types of LC specimens cured under water until testing or cured under water for seven days and subsequently in a controlled climate chamber at 20 °C/65% relative humidity [64]. The conversion factor for NC (0.96) does not distinguish between various specimen types or sizes. The impact of curing determined for LC depends on the type of specimen and the age of testing [117]. Cylinders do not exhibit any effect at 28 d, but at 90 d. Strength of cubes is more sensitive against curing conditions due to the faster drying of the corners. Here, a clear effect can be observed at 28 d. Strength values of small cubes (100 mm) are more sensitive than those measured on bigger cubes (150 mm and 200 mm) as the effect of corner drying on the cube is more severe for smaller samples. At 90 d, the curing impact leads to a conversion factor of 0.96 for both LC specimen shapes, which is the one used for NC.

## 5. Application Areas of LC and ILC Depending on Strength, Density and Thermal Conductivity 

### 5.1. Application Areas of LC

**Figure 8:**
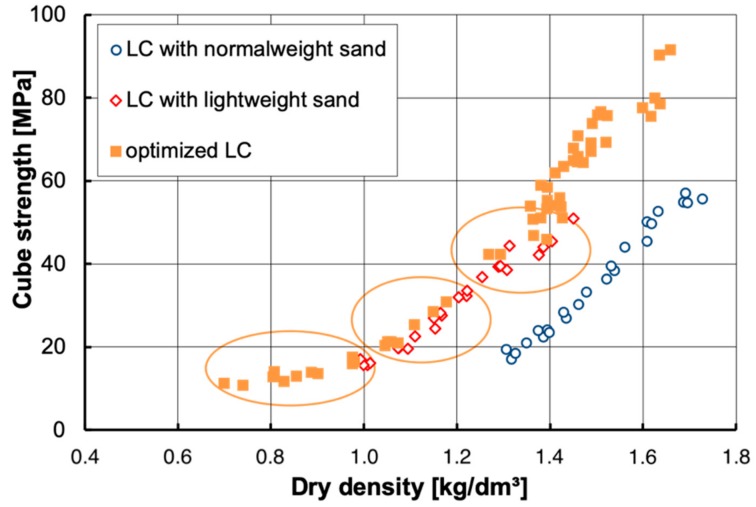
Correlation between 28-day cube strength and dry density for lightweight concretes with different compositions [6].

LC is suitable for a wide range of applications due to its versatility. Much information is available for LC made with coarse LWA and normal weight sand since it is quite common worldwide. The most interesting and challenging LC structures are bridges [5,118,119,120,121,122,123,124,125,126,127,128,129] and offshore platforms [13,130,131,132]. A broad range of LC projects is described in [41,113]. In the following, the focus will be on LC made with coarse LWA and lightweight sand as they provide additionally interesting thermal insulation properties. For this approach, three fields are identified in Figure 8 [6]. A similar designation is given by Dilli et al. [133]. Further information on the correlation between compressive strength and density of LC covering a wide range of mix designs and LWA types are available from databases [33,134,135].

- A concrete dry density in the range between 1.3 and 1.6 kg/dm^3^ is aimed for highly stressed facades of office buildings with many and wide window and door openings. They demand for a higher compressive strength in combination with a reduced thermal conductivity of the LC.
- Less stressed facades with higher requirements regarding thermal insulation are built with LC densities in the range between 1.0 and 1.3 kg/dm^3^. These walls often have a thickness of 50 cm in order to fulfill legal requirements regarding building physics.
- The third field of LC with a dry density < 1.0 kg/dm³ represents the most innovative part these days in central Europe. These very light LC offer the best thermal insulation for monolithic concrete and are increasingly used for exclusive private houses. The architect Gartmann developed a fair faced LC for the monolithic exterior walls of his private house [136]. His idea has been adopted by other architects and developed further by concrete technologists. Since the thermal insulation requirements demand rather thick walls, the achievable strength usually provides sufficient load bearing capacity for single- or two-story houses.

### 5.2. Extending the Application Area of ILC

A new approach is needed since building physics requirements ask for thermal insulation properties, which cannot be accomplished within the limits defined in the standards for structural lightweight concrete. Some ILC exhibit a dry concrete density below 800 kg/m^3^ and thus are no longer covered by existing LC standards [48]. Schlaich and his co-workers (Technical University Berlin, Germany) have extrapolated the application rules for LC below the existing limits for strength values and densities in DIN EN 1992 and established design rules for infra-lightweight concretes (ILC) [137,138,139]. 

**Figure 9:**
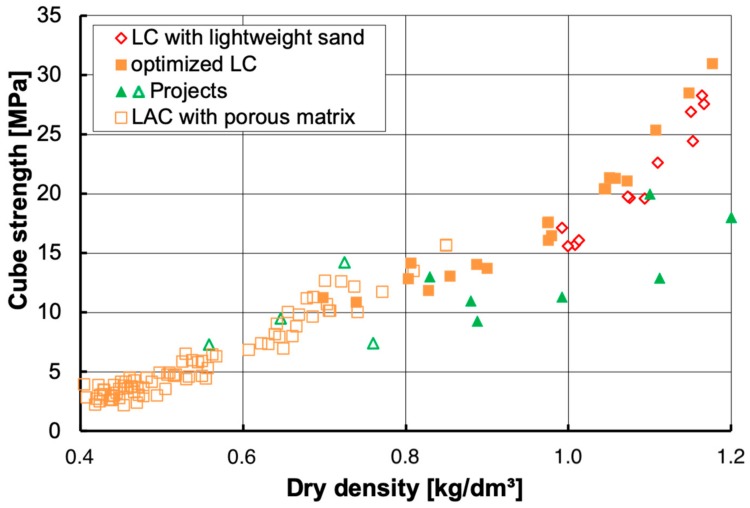
Correlation between 28-day cube strength and dry density for lightweight concretes (LC) with lightweight sand [143], lightweight aggregate concrete (LAC) with porous matrix different compositions [43], data for two ILC projects [18,48] and most recent LAC-based ILC projects [49].

Recently, another approach to extend the application range of ILC was started at Universität der Bundeswehr München, Germany. Since the mix design of ILC is rather close to LAC, due to the high air void content (see Section 2.2), it was self-evident to adopt the relevant standard EN 1520 [42] for cast-in-place concrete. The advantage of using EN 1520 as basis for structural design is the possibility to cover even dry densities as low as 400 kg/m^3^. At the same time, mean strength can be reduced to less than 4 MPa which corresponds with strength class LAC 2 [42]. Figure 9 is a blow-up of Figure 8 and displays additionally the values of executed projects with very light LC and those of the first two ILC projects [18,48]. It shows also the average values obtained within the first two projects following the new LAC-based approach in comparison with LAC data published earlier [43]. The first two ILC projects that follow this LAC-based approach achieved dry densities of 600 kg/m^3^ and less on site and strength values suitable for LAC 4 [49]. 

**Figure 10:**
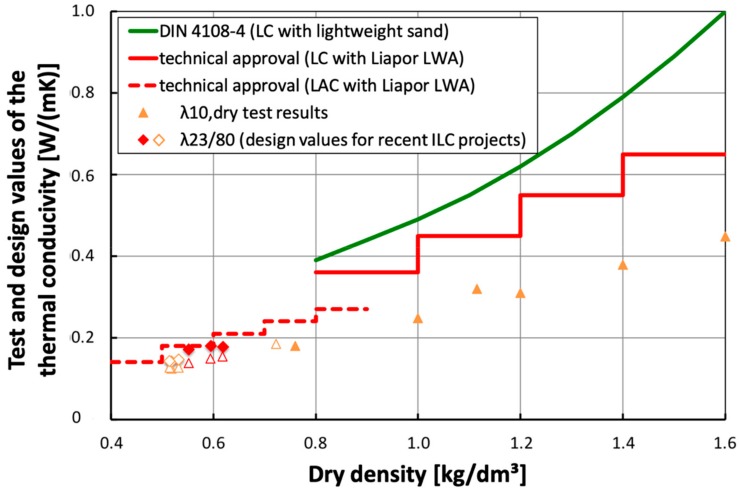
Correlation between thermal conductivity and dry density for LC, LAC taken from technical approvals [140,141], data published for several LC and two ILC projects [18,48] as well as for most recent LAC-based ILC projects [49].

The new LAC based approach is very successful with respect to the thermal insulation properties of the LAC based ILC. Figure 10 displays measured and design values of the thermal conductivity of projects as well as values laid down in technical approvals [140,141]. The data of the new ILCs will open new opportunities for the producers in an interesting market. An overview with thermal conductivity values of LC and a focus on dry densities above 1400 kg/m^3^ is given in [142].

**Figure 11:**
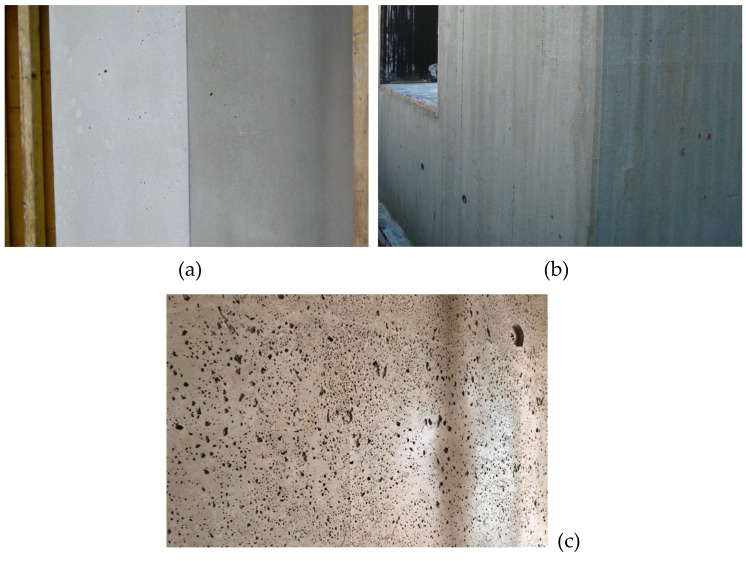
Surface texture of executed ILC projects. Perfectly smooth surface (**a**); rough saw surface (**b**); porous (vivid) surface (**c**) (courtesy: Björn Callsen and Werner Rothenbacher)

Another very important challenge for ILC is maintaining the appearance of a fair faced concrete which is mandatory for meeting the expectations of future house owners and architects. It is not possible to classify the surface quality according to the same technical and design criteria as applied for NC, such as defined in [144]. Although ILC clients expect or even demand a rather “vivid” surface texture [143] (Figure 11), special considerations are necessary regarding a suitable conformity concept and durability for ILC. Thus, ILC requires a project-related approval or a technical approval.

**Figure 12:**
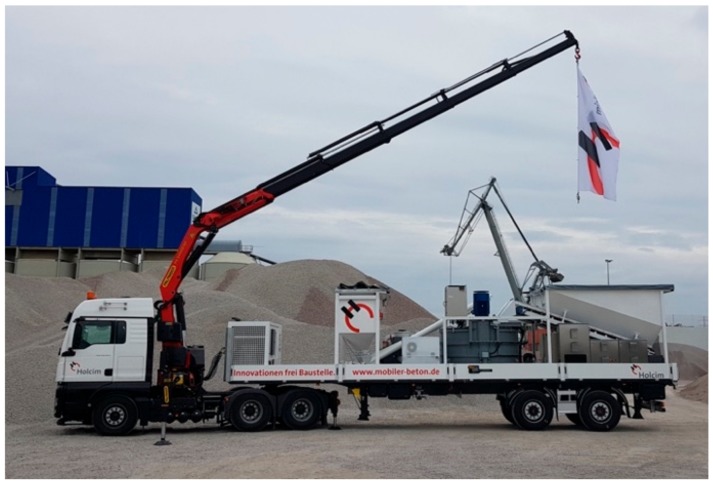
Truck-mounted concrete plant for ILC (courtesy: Björn Callsen).

Currently there are two deviating ILC approaches under investigation in close cooperation with concrete producers. The first one is a conventional course of action: the ILC is produced in a concrete plant, delivered in a conventional mixing truck and placed on site with a concrete bucket. Quite often, architects who are interested in using ILC for their projects fail finding a concrete producer willing or capable to provide ILC. Since the request for LC is low in most regions, common concrete plants are not equipped for the production of LC or even ILC or suffer from a lack of silo capacity. Both dilemma can be solved with the second approach: the ILC is produced under controlled conditions on site by means of a tailored truck-mounted concrete plant (Figure 12).

**Figure 13:**
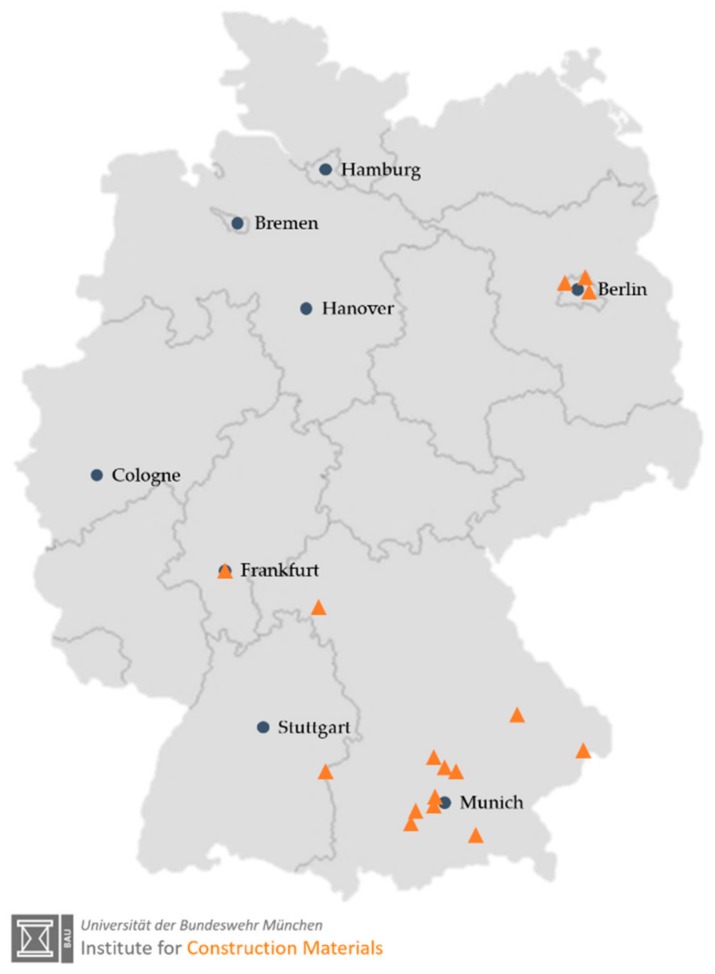
Map of Germany showing locations where ILC-projects have been realized until end 2019.

Despite the lack of a basis for production and execution rules for lightweight concretes below a dry density of 800 kg/m³, the increased interest of clients and architects has led to the construction of various single and multi-family houses. Figure 13 shows a map that gives the locations of ILC construction projects carried out in Germany until the end of 2019. It was necessary to obtain a project-related approval for these projects. The experience from construction and ongoing research projects can form the basis for the further development of design criteria and promote the establishment of very light LC in new fields of application. Future work will focus on the development ILC with competitive thermal insulation properties while maintaining appropriate design and durability specifications. 

## 6. Conclusions

Lightweight concrete is an extremely versatile material, which can be used for a wide range of applications. Although lightweight concrete has been in use for two millennia, there are still uncertainties, which were addressed in this review paper. Clear definitions were given for the different types of lightweight concrete and information was provided about crucial topics regarding lightweight aggregate properties as well as mix design, testing and classification of structural lightweight concrete. Several issues represent key information in the state-of-the-art of lightweight concrete. 

Infra-lightweight concretes (ILC) constitute an innovative development with very low dry densities (<800 kg/m³) and respectable thermal insulation properties. ILC is the answer to a new and unexploited area of application. Future research is an essential precondition for the development of a reliable design and construction methodology to ensure production quality and to prepare a European standardization concept.

## Figures and Tables

**Table 1:** Compressive strength and splitting tensile strength requirements for LWA [24].

Calculated Equilibrium Density max, kg/m^3^	Average 28-day Splitting Tensile Strength, min, MPa	Average 28-day Compressive Strength, min, MPa
All Lightweight Aggregate		
1760	2.2	28
1680	2.1	21
1600	2.0	17
Combination of Normal Weight and Lightweight Aggregate		
1840	2.3	28
1760	3.1	21
1680	2.1	17
